# Unveiling tracheo-oesophageal fistula: The crucial role of imaging in the diagnosis and management

**DOI:** 10.4102/sajr.v29i1.3216

**Published:** 2025-09-23

**Authors:** Poonam Sherwani, Nivedita Sharma, Rajat Piplani, Ekakshi Varshney, Sumit Kumar

**Affiliations:** 1 Department of Diagnostic and Interventional Radiology, All India Institute of Medical Sciences (AIIMS), Rishikesh, India; 2 Department of Pediatric Surgery, All India Institute of Medical Sciences (AIIMS), Rishikesh, India; 3 Department of Radiology and Imaging, Govind Ballabh Pant Institute of Postgraduate Medical Education and Research (GPIMER), Delhi

**Keywords:** contrast oesophagogram, oesophageal stricture, tracheo-oesophageal fistula, ultrashort echo time MRI, VACTERL association, fistulous, foregut

## Abstract

Tracheo-oesophageal fistula (TOF) is a significant congenital anomaly characterised by an abnormal communication between the trachea and the oesophagus. Prompt diagnosis and surgical intervention are crucial, but long-term outcomes depend on accurate imaging and postoperative monitoring. The article discusses the embryological basis, clinical presentation and various types of TOF with their imaging techniques. Immediate and long-term postoperative complications are also discussed. Recent advances such as Ultrashort Echo Time (UTE), MRI and the Oesophageal Anastomotic Stricture Index (OASI) have expanded the capabilities of imaging in predicting surgical outcomes and guiding follow-up.

**Contribution:**

This review highlights the pivotal role of imaging in the diagnosis, classification, surgical planning and follow-up of TOF, focusing on current and emerging modalities.

## Introduction

Congenital tracheo-oesophageal fistula (TOF) is one of the most frequently encountered congenital abnormalities in common clinical practice. The term describes a condition where there is a persistent fistulous communication between the trachea and the oesophagus. Infants usually present with breathing and feeding difficulties with an associated risk of aspiration. Various imaging modalities such as radiographs, contrast swallow studies, CT and MRI are required for the diagnosis as well as to look for complications; however, the choice of modality should be individualised based on the clinical scenario. In this review article, the role of various imaging modalities will be described along with their benefits and drawbacks.

### Embryology and types of tracheo-oesophageal fistula

During early gestation, the lung bud arises from the ventral aspect of a single embryological foregut. Subsequently, the trachea and oesophagus develop distally through a process of septation, wherein the trachea forms as a diverticulum from the foregut. A mesenchymal septum progressively separates the trachea from the oesophagus. Abnormal posterior displacement of this septum results in incomplete separation of the two structures, leading to a persistent TOF.^1,2^

This process is tightly regulated in both spatial and temporal dimensions by the notochord, which modulates Sonic Hedgehog (SHH) gene expression. Disruption in this signalling pathway can result in defective tracheo-oesophageal separation, often in association with other anomalies comprising the VACTERL spectrum (vertebral, anal, cardiac, tracheo-oesophageal, renal and limb anomalies).^1^

Congenital TOFs are classified into types A to E based on the Gross classification:

- **Type A:** Pure oesophageal atresia (OA) without a fistula.
- **Type B:** OA with a proximal TOF.
- **Type C:** OA with a distal TOF (most common type).
- **Type D:** OA with both proximal and distal fistulas.
- **Type E (H-type or N-type):** TOF without OA.^3^ A fistulous tract connects the trachea and oesophagus without associated OA, often resembling the shape of the letter “H” or “N” on imaging.

**FIGURE 1:**
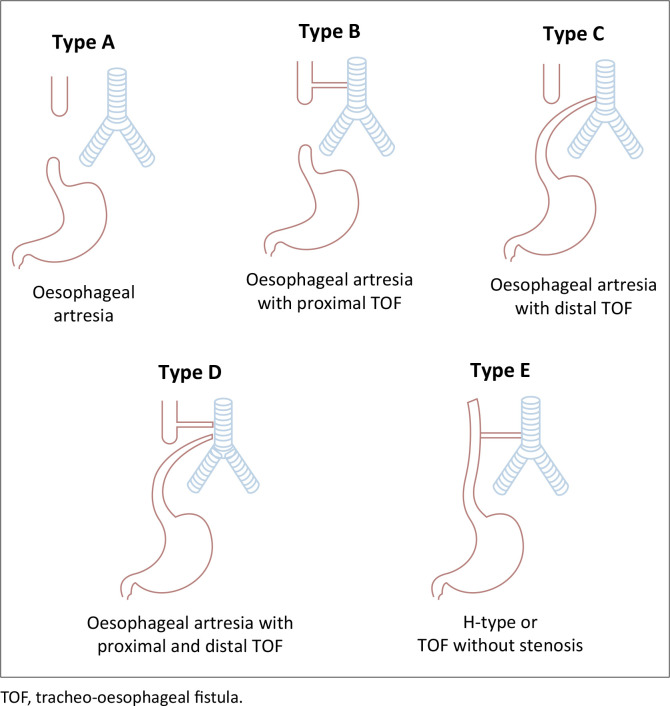
Gross diagrammatic representation of types of tracheo-oesophageal fistula.

From a surgical perspective, types A and B are particularly challenging because of the presence of a long gap between the proximal and distal oesophageal segments and are thus referred to as long-gap OA. A schematic representation of the various TOF types is illustrated in Figure 1.

## Clinical presentation

The classic presentation of OA at birth is an infant who exhibits excessive mucous and drooling because of saliva pooling in the blind-ending upper oesophageal pouch. Infants displaying these signs should not be fed until OA has been definitively excluded. Additional clinical indicators include tachypnoea, coughing and choking during attempts to feed.

Approximately one-third of infants with OA are born prematurely, and maternal polyhydramnios is reported in about 30% of cases – this incidence is notably higher in infants without a distal TOF. The presence of associated anomalies known to occur with OA should raise clinical suspicion. For instance, in cases with polyhydramnios or anorectal malformations, the patency of the oesophagus should be assessed early by attempting to pass an orogastric tube shortly after birth.^1^

The incidence of TOF is 2.9 per 10 000 worldwide. It is generally diagnosed when the clinician is unable to insert a feeding tube. Diagnosis of TOF is usually made clinically, however, imaging is required in doubtful cases and in H-shaped fistula. Radiographs are the preliminary investigation performed to look for air in the stomach and associated anomalies in the spine. When the components of the VACTREL complex are detected, a detailed and thorough investigation should be made to evaluate for the rest, which includes a meticulous clinical examination and necessary imaging.

Air in the stomach is present when there is fistulous communication between the lower oesophagus and trachea (Types C, D and E), in which cases the clinical diagnosis is usually straightforward. Radiological investigations play an important role not only in determining the type of TOF and aiding its management but also in diagnosing the postoperative complications of TOF repair, including oesophageal strictures, recurrence of TOF, tracheomalacia and reflux.

## Imaging

### Antenatal examination

Radiologically, TOF can be determined antenatally through ultrasound at approximately the 20th week of gestation. The demonstration of a dilated oesophagus or pouch oesophagus on swallowing is the most important finding and has 100% positive predictive value.^4^

### Postnatal examination

#### Radiographs

**FIGURE 2:**
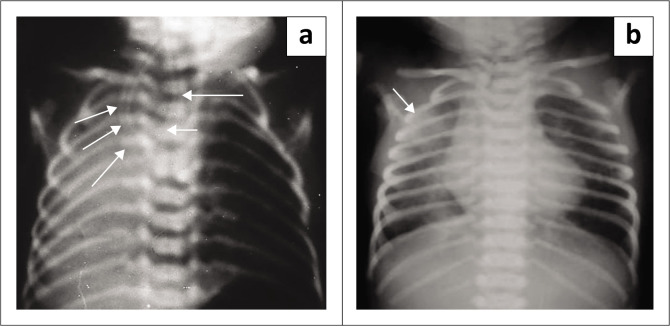
(a) Frontal radiograph of a neonate depicting the coiled nasogastric tube (white arrows), multiple vertebral anomalies, and an opaque right hemithorax with ipsilateral mediastinal shift suggestive of lung hypoplasia. (b) Another neonate in whom there was difficulty in passing the nasogastric tube showed aspiration-related changes in the right upper lobe (white arrow).

The initial investigation performed is usually a radiograph, which shows a dilated segment of the oesophagus, otherwise known as a pouch, which is seen on anteroposterior and lateral views. There are usually three imaging features to be reported in a suspected case of TOF:

1. Presence of a TOF: Inability to pass a 9–10 French nasogastric tube for the length of approximately 9–10 cm from the gums.
2. Presence or absence of gas in the abdomen – The presence of gas in the abdomen suggests a distal TOF (types C–E). Absence of gas in the abdomen suggests type A or B.
3. Associated anomalies (look for other components of VACTREL complex) as shown in Figure 2.

In indeterminate cases, non-ionic contrast may be injected into the pouch to confirm the diagnosis and evaluate the proximal segment, however, there is risk of aspiration.

#### Contrast swallows

**FIGURE 3:**
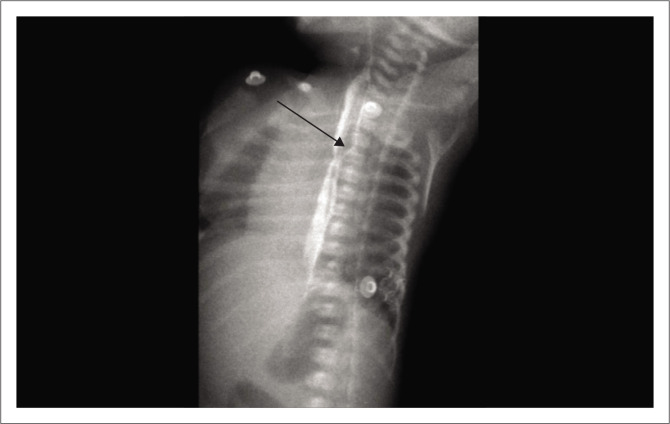
Contrast swallow lateral oblique view depicting the fistulous communication (N-type) between the oesophagus and trachea (black arrow).

An oesophagogram with water-soluble contrast is the ideal investigation for TOF. Single contrast oesophagram using pulsed digital fluoroscopy is performed in the right lateral and right anterior oblique position to achieve complete distension of the oesophagus (Figure 3).

If a suspected fistula is not detected using a contrast swallow study, an H-shaped TOF may be considered. To investigate this, a feeding catheter is inserted into the oesophagus up to the lower oesophagus and then gradually withdrawn while injecting the contrast agent under fluoroscopy.

Proper technique and various precautions are required to demonstrate the H or N-shaped fistula.

1. The child should be placed either prone or semi-prone with a mild head-down position to demonstrate the fistula and to avoid aspiration.
2. Small amounts of contrast should be infused slowly to avoid missing the subtle fistula and to avoid injecting the contrast into the airway.
3. Continuous real-time fluoroscopic imaging is required, with imaging focused on the cervical and high thoracic regions, as most fistulas are above the level of the carina.

However, contrast tracts may also be observed in cases of a tracheolaryngeal cleft or aspiration. Distinguishing TOF from these conditions is crucial because of their differing management approaches. A tracheolaryngeal cleft is characterised by an abnormal connection between the larynx and trachea.

**FIGURE 4:**
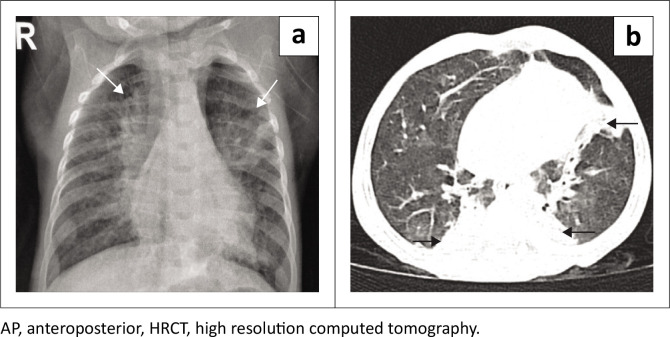
(a) AP chest radiograph demonstrates aspiration changes in the upper zones bilaterally. (b) HRCT thorax of the same patient confirms upper lobe aspiration changes.

**FIGURE 5:**
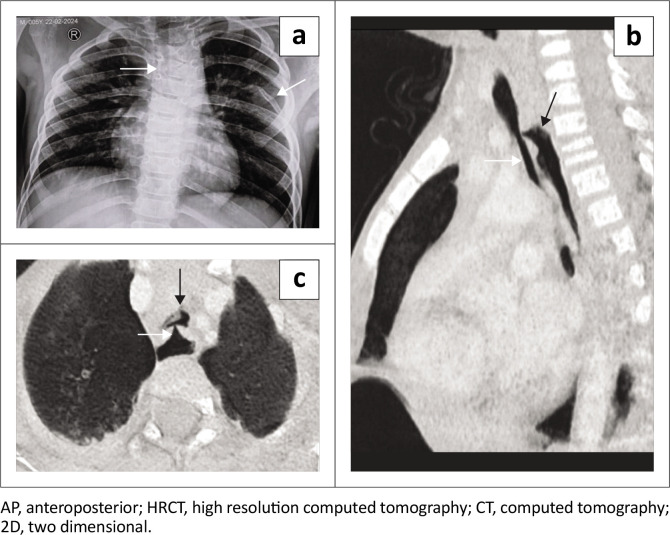
A 3.5-year-old female presented with fast breathing, feeding difficulties and regurgitation of food. (a) AP chest radiograph demonstrates vertebral and rib anomalies (white arrows). Oral swallow study failed to demonstrate the presence of a fistula. Subsequently, HRCT thorax confirmed the presence of an H-type fistula: arrows in (b) sagittal reformatted chest CT image of the trachea (white arrow) and oesophagus (black arrow), and (c) the axial MinIP image of the trachea (black arrow) and fistulous communication (white arrow).

CT can be used as a solving tool when a fistulous communication is not seen on fluoroscopy and to detect aspiration changes (Figure 4). Various software, such as Minimum intensity projection (MinIP), Maximum intensity projection (MIP), Multiplanar reformation (MPR) and virtual bronchoscopy, can be used to identify the fistula (Figure 5). The gap between the oesophageal pouches can be evaluated on a single image.

## Surgical management

The treatment plan for each baby should be individualised. The surgical options for OA or TOF repair include open thoracotomy or video-assisted thoracoscopic surgery.^5^ In either operation, the steps involved are similar.

- Type A, pure OA without TOF presents with a gasless abdomen on babygram and requires cervical oesophagostomy and feeding gastrostomy.
- Type B, OA with proximal TOF is very rare (< 1%) and requires a cervical approach for fistula ligation along with a feeding gastrostomy. A preoperative bronchoscopy to delineate the fistula is also useful in these patients.
- Type C, OA with distal TOF is the most common type and requires a right-sided posterior-lateral thoracotomy using an extra-pleural approach (preferred) with fistula ligation followed by end-to-end oesophageal anastomosis. For long-gap OA, many oesophageal lengthening procedures have been published in the literature.^6^ However, primary cervical oesophagostomy and feeding gastrostomy are preferred. This can be followed by oesophageal replacement with stomach (gastric pull-up or gastric tube), jejunal or colonic pull-through procedures after a few months.
- Type D, OA may require both a cervical approach for fistula ligation and thoracotomy with primary oesophageal anastomosis.
- Type E, TOF without OA or H-type fistula needs only fistula ligation, either through the cervical or thoracotomy approach. The surgical approach is dependent upon the vertebral body level of the TOF as demonstrated by either oesophagogram or endoscopic examination. A transcervical approach is preferred for any fistula at the thoracic inlet or higher. A thoracotomy is generally required only for fistulas below the level of the second thoracic vertebral body.^7^

## Complications

With the advent of paediatric critical care, there is a significant reduction in the incidence of postoperative mortality following TOF surgery, with a survival rate above ~90%.^8^ However, a few postoperative complications can occur following repairs, including anastomotic leak, stricture and recurrent fistula formation. Apart from these, there are long-term complications that can occur after surgery, namely gastro-oesophageal reflux disease (GORD), oesophagitis, dysphagia, tracheomalacia, vocal cord disorders and risk of oesophageal malignancy.

### Postoperative complications

#### Anastomotic leak

**FIGURE 6:**
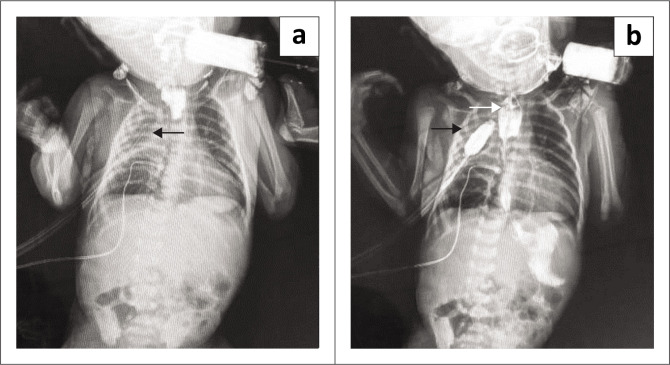
Postoperative tracheo-oesophageal fistula complication. (a) Contrast study depicting a right loculated hydropneumothorax with a chest tube in situ (black arrow). (b) Dilated upper oesophagus (white arrow) with extravasation of contrast into the right pleural cavity (black arrow).

Anastomotic leaks are one of the most common postoperative complications wherein an anastomotic disruption leads to leakage of contents into the mediastinum with the development of a critical fluid collection or pneumothorax.^8^ Leaks may be classified as minor or major based on the extent of anastomotic disruption.^8^ An oesophagogram is an effective diagnostic tool for detecting leaks, as contrast material can be observed escaping from the anastomosis into the mediastinum (Figure 6). Small leaks, characterised by minimal contrast leakage into the mediastinum, generally do not require intervention. Leaks are more common in long gap OA and are influenced by several factors, including the level of tension at the anastomotic site, the execution of the myotomy,^9^ use of braided silk sutures and prolonged periods of non-invasive ventilation.^10^

#### Anastomotic stricture

**FIGURE 7:**
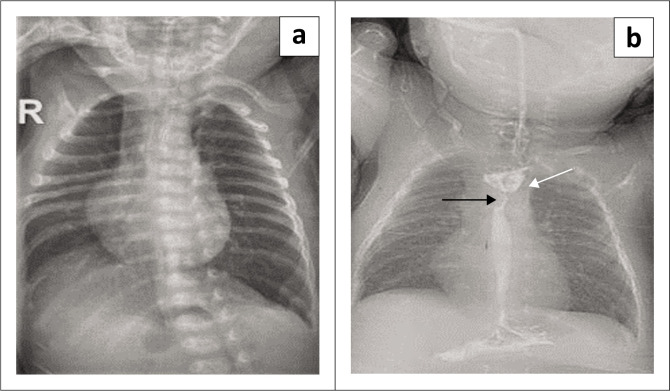
Postoperative tracheo-oesophageal fistula complication. The child presented with difficulty in feeding and tolerating a semisolid diet. (a) The chest radiograph revealed hyperinflated lungs with flattened diaphragmatic domes. (b) Contrast was administered via the mouth, revealing a stricture in the mid-thoracic oesophagus (black arrow) and a dilated proximal oesophagus (white arrow).

The incidence of stricture after TOF repair is estimated to range from 10% to 59%.^11^ It signifies a narrowing at the anastomotic site and is associated with difficulties in feeding, drooling, vomiting and the inability to gain weight. Although endoscopy can be used to identify a stricture, diagnosis can also be made non-invasively through an oesophagogram (Figure 7), along with the added benefit of determining measurements. Various risk factors include oesophageal tension, anastomotic leak and gastroesophageal reflux and it is also more common in long-gap OA because of staged procedures. Another predisposing factor is the use of magnets during surgery, known as magnamosis, needed to achieve oesophageal continuity. The Oesophageal Anastomotic Stricture Index (OASI) is useful to ascertain the degree of stenosis.^11^ The OASI is calculated for upper (U-OASI) (Equation 1) and lower (L-OASI) (Equation 2) pouches, using the stricture diameter (d) and the pouch diameter (D):


$$\text{U}-\text{OASI}:\frac{\text{Lateral d}/\text{D}+\text{Anteroposterior d}/\text{D}}{1}$$ [Eqn 1]

$$\text{L}-\text{OASI}:\frac{\text{Lateral d}/\text{D}+\text{Anteroposterior d}/\text{D}}{2}$$ [Eqn 2]

d: Stricture diameter; D: Pouch diameter.

A ratio of 0.25 indicates that the diameter at the anastomosis is 25% of the diameter of the patient’s normal oesophagus. Smaller ratios indicate even tighter strictures.^12^

However, during the immediate postoperative period, narrowing on the oesophagogram should not be labelled a stricture, as the appearance may related to temporary oedematous changes. Transit oedema resolves within 2 to 4 weeks, and hence stricture should be considered if there is persistent narrowing beyond 4 weeks or there are persistent clinical symptoms such as dysphagia or feed intolerance.^9^ The development of anastomotic stricture is attributed to numerous causes which include anastomotic tension, anastomotic leak, gastro-oesophageal reflux (GOR),^13^ vascular compromise, ischemia at the anastomotic site,^14^ staged repair of TOF,^15^ use of braided silk sutures and magnamosis (achieving anastomosis using magnetic devices).^16^

#### Recurrent tracheo-oesophageal fistula

The incidence of recurrent TOF has been stated to range from 3% to 15%.^17^ Multimodality imaging, with oesophagogram, bronchoscopy, oesophagoscopy, contrast swallow and CT scan, is recommended when suspecting recurrent TOF, as it can be easily missed on single-modality imaging because of postoperative inflammation and oedema. Recurrent TOF should be differentiated from postoperatively acquired TOF or undiagnosed congenital TOF. Smithers et al. classified postoperative ‘recurrent’ TOF into three categories: congenital TOF (congenital), recurrent TOF and acquired TOF. Congenital TOFs are those that persist after surgery because either they were missed on imaging or an incomplete repair was performed. These are typically present immediately after the surgery. Recurrent TOF occurs in the same location as the repaired primary TOF. On the other hand, acquired TOF develops at a different site along a new pathway.^18^ Recurrent TOF has been related to anastomotic leaks, pressure necrosis by clips or sutures, repeated oesophageal dilatations and infection.^18,19^

### Long-term effects

Apart from the postoperative complications discussed earlier, multiple long-term effects manifest over weeks and months, and can cause significant patient distress.

#### Dysphagia

Dysphagia is one of the most frequently encountered complaints after TOF surgery. In children, many develop adaptive feeding behaviours; some may also suffer from failure to thrive and aspiration in the long term.^20^ It is easily diagnosed using manometry which shows reduced and weak peristalsis with aberrant contraction patterns.^21^ It is strongly associated with GOR. A fluoroscopic oral swallow study can also be used to visualise the impaired deglutition phases in real time.^22^

#### Gastro-oesophageal reflux

Regurgitation of gastric contents is another commonly encountered complaint linked to anastomotic tension, incompetent lower oesophageal sphincter, injury to the vagus nerve during surgery and a widened intra-abdominal hiatus.^23^ Gastro-oesophageal reflux can be diagnosed accurately via pH impedance and upper gastrointestinal endoscopy with biopsy to rule out eosinophilic oesophagitis. It is refractory to medical treatment and frequently requires surgery.^20^

#### Tracheomalacia

Tracheomalacia is defined as collapse of the trachea during expiration and is highly prevalent in patients with congenital TOF/OA. It is also a complication of TOF repair, occurring secondary to a weak tracheal wall, which is in turn is caused by postoperative changes, prolonged intubation and anastomotic strictures.^24^ It is diagnosed using laryngotracheobronchoscopy. A dynamic CT scan (end-inhalation and end-exhalation) can be used to describe the location and the extent of wall collapse.

#### Vocal cord dysfunction

The highest prevalence of postoperative vocal cord dysfunction is seen in patients with H-type TOF, and the lowest in Type C TOF.^25,26^ It occurs because of injury to the vagus or recurrent laryngeal nerves. Vocal cord injury is a clinical diagnosis and, in most cases, it resolves spontaneously.^25^

#### Oesophageal malignancy

Few patients with OA repair have been shown to develop oesophageal malignancies later in life secondary to GORD and associated mucosal dysplasia. Histopathology usually shows squamous cell carcinoma, but a case of a patient with colonic interposition developing colorectal carcinoma has also been documented. Endoscopic surveillance of OA patients is therefore recommended for early detection of any lesions.^27^

## Recent advances in imaging of TOF

Nara et al.^28^ highlighted the emerging role of Ultrashort Echo Time (UTE) MRI as a valuable tool in the preoperative assessment of OA and TOF. This advanced imaging technique provides detailed anatomical visualisation, which is crucial for surgical planning. In their study, UTE MRI was used to measure the widest diameter of the proximal oesophageal pouch and the angle of tracheal deviation. Patients with more pronounced proximal oesophageal dilation and greater tracheal deviation were found to require prolonged postoperative respiratory support. These findings suggest that UTE MRI not only aids in anatomical delineation but also offers potential in predicting postoperative outcomes, representing a significant advancement in the management of OA/TOF.

## Conclusion

Tracheo-oesophageal fistula is a complex congenital anomaly that necessitates a thorough understanding of embryology, clinical presentation and the nuances of diagnostic imaging. The timely identification and classification of TOF are essential for appropriate surgical planning and postoperative care. Imaging plays a central role not only in the diagnosis but also in monitoring for postoperative complications, including anastomotic strictures, leaks, recurrent fistulas and long-term sequelae such as GORD and dysphagia. Recent advances, particularly in MRI techniques, such as UTE MRI, show a promise in enhancing anatomical assessment and predicting clinical outcomes. Continued refinement in imaging and surgical approaches will be pivotal in improving patient outcomes and quality of life in children with TOF.

## Teaching points

- TOF is a common congenital anomaly and is frequently associated with other abnormalities, especially those in the VACTERL spectrum.
- Imaging is critical for diagnosis, classification, surgical planning and identification of postoperative complications.
- Radiographs and contrast studies are the first-line investigations, while CT and advanced MRI techniques (e.g., UTE MRI) offer detailed anatomical information.
- Postoperative complications include anastomotic leaks, stricture formation, recurrent TOF, GORD, tracheomalacia and, rarely, malignancy.
- Newer indices like OASI help quantify strictures, while innovations like magnamosis change the management of long-gap OA.
- Long-term surveillance is necessary because of potential complications, including oesophageal malignancy in adulthood.
